# Imaging Sonar Detection and Occlusion-Corrected Abundance Estimation in High-Density Aquaculture

**DOI:** 10.3390/s26206387

**Published:** 2026-10-09

**Authors:** Tianhao Zhao, Xiaolong Zhang, Yuhang He, He Ma, Ying Liu, Zhihong Ma

**Affiliations:** 1 College of Biosystems Engineering and Food Science, Zhejiang University, Hangzhou 310058, China; zhaotianhao01@zju.edu.cn (T.Z.);; 2 College of Marine Technology and Environment, Dalian Ocean University, Dalian 116023, China; 3 College of Fisheries and Life Science, Dalian Ocean University, Dalian 116023, China

**Keywords:** imaging sonar, Poisson distribution, spatial extrapolation, abundance estimation, recirculating aquaculture system

## Abstract

Real-time numerical abundance monitoring in high-density recirculating aquaculture systems (RASs) faces severe bottlenecks due to target occlusion saturation and spatial sampling mismatch. This study establishes an acoustic–statistical framework coupling deep learning detection with spatial statistical theory across sequential detection, correction, and scale expansion stages. To resolve systematic counting underestimation caused by target occlusion and acoustic shadowing, a Poisson coverage model incorporating morphological eccentricity (e) is formulated to statistically correct visual detection biases. To extrapolate localized acoustic observations to whole-pond fish abundance, a spatial-scale expansion model is developed based on localized roaming behavior and spatial stratification, parameterizing horizontal (α) and vertical (ν) distribution coefficients. Under controlled density gradients (20–100 individuals), the framework achieves a minimum relative estimation error of 9.58%. In commercial pond validation trials (6000–6500 individuals), post-correction global relative errors drop to 1.38–16.43%, substantially improving upon uncorrected visual detections (38.77–53.12%). This framework offers an automated, frame-level acoustic sensing workflow for monitoring fish abundance, providing foundational numerical data for feeding optimization and stock tracking in intensive aquaculture facilities.

## 1. Introduction

Fish are a crucial protein source in global food security. Accurate population quantification is therefore essential for optimizing feeding, regulating water quality, and ensuring production efficiency. In intensive aquaculture, precise stock abundance data determine daily feed intake, preventing underfeeding or overfeeding. At the macro-level, stock assessments underpin sustainable production, conservation strategies, and optimal resource allocation. The widespread adoption of intensive recirculating aquaculture systems (RASs) has rendered traditional manual statistical methods inadequate due to their high invasiveness, low efficiency, and large errors, failing to meet modern demands for dynamic and precise management.

Acoustic techniques offer a non-invasive alternative for monitoring stock abundance in turbid or dark environments, developing along two primary approaches [1,2]. The target strength (TS) analysis method counts individual fish by identifying echo signals, making it suitable for low-density populations and size estimation. Recent studies have validated strong linear correlations between lateral TS values and physical body metrics under free-swimming conditions [3]. However, this method is limited by sonar resolution, where fish overlap often leads to missed detections. Furthermore, the conversion from TS to weight requires complex calibration because individual echo structures fluctuate significantly with fish yaw distributions, depth, and physiological variations [4,5]. Another method is the volume backscattering strength ($S_{v}$), which directly estimates biomass from echo intensity and offers high computational efficiency in high-density scenarios. However, its accuracy depends on empirical TS coefficients that are sensitive to species, size, and posture, potentially leading to errors of up to 30% in dense aggregations.

High-resolution imaging sonar overcomes some of these limitations by generating near-video-quality acoustic images in turbid, low-light conditions for visual identification and tracking [6]. Nevertheless, practical applications are still limited by acoustic shadowing, severe background noise, and a limited detection range (2–40 m), making it difficult to fully cover large ponds. These interferences necessitate robust background elimination, filtering, and morphological extraction to isolate target contours or shadow zones [7,8]. Furthermore, overlapping fish in dense aggregations cause missed detections and double-counting. Conventional TS models based on empirical intensity-length relationships cannot be directly applied to pixel-level target detection, limiting abundance inversion accuracy.

To address these challenges, two main technical avenues have been explored. First, deep learning object detection algorithms, especially the YOLO series and convolutional networks, have been widely applied to acoustic image analysis for their real-time accuracy [9,10]. Related studies have employed point supervision, semi-supervised density proxies, and transformer regression heads. These enhancements improve target localization, minimize background noise, and handle fish deformations in high-density scenes [11,12,13]. Second, statistical models such as the Poisson distribution or random encounter frameworks have been applied to density estimation, confirming the effectiveness of probabilistic modeling in population quantification without individual tracking requirements [14,15].

Existing acoustic monitoring methodologies face two critical physical and geometric constraints in intensive aquaculture. First, visual and acoustic information suffers from severe saturation and signal degradation under high-density conditions, where extreme target occlusion and acoustic shadowing cause standard optical and acoustic detection algorithms to fail. Second, a severe spatial mismatch persists between two-dimensional (2D) cross-sectional sampling and three-dimensional (3D) non-uniform population distributions. Because imaging sonar scans only a limited 2D sector while fish exhibit heterogeneous spatial utilization, extrapolating local acoustic observations to global population size without geometric calibration induces substantial structural distortion. Current isolated paradigms—whether purely vision-based or strictly statistical—fail to resolve these coupled physical-geometric constraints [16], leaving stock abundance estimation restricted to uncalibrated localized boundaries.

To address these coupled physical and geometric constraints, this study develops an integrated acoustic–statistical framework coupling deep learning detection with spatial point process modeling across sequential stages of detection, correction, and scale expansion. The pipeline first executes real-time target localization and multi-frame kinematic tracking on raw sonar streams. Subsequently, a micro-scale Poisson coverage model rectifies counting underestimation induced by mutual body occlusion and acoustic shadowing within the 2D acoustic slice. Finally, horizontal (α) and vertical (ν) spatial distribution coefficients derived from localized roaming behavior extrapolate localized cross-sectional counts to whole-pond numerical abundance. Validation was conducted using juvenile largemouth bass (*Micropterus salmoides*) across density gradients and commercial recirculating aquaculture facilities, alongside qualitative verification on golden pompano (*Trachinotus ovatus*) in offshore sea cages.

Pioneering sonar studies in cage aquaculture have primarily focused on 2D attention-enhanced detection architectures [17], vertical dispersion clustering [18], or localized behavioral tracking [19]. In contrast, the distinctive contribution of this work lies in two core aspects. First, we establish a closed-form micro-scale Poisson coverage model that statistically rectifies target overlapping and shadowing saturation without relying on multi-stage regressors. Second, we formulate a two-level spatial expansion scheme coupling horizontal roaming (α) and vertical stratification (ν) coefficients, bridging the dimensional gap between localized 2D acoustic slices and 3D whole-pond abundance.

## 2. Materials and Methods

### 2.1. Equipment and Dataset

This study employed the D750 dual-frequency imaging sonar system manufactured by Shanghai Hanjie Technology Co., Ltd. (Shanghai, China). Its main technical parameters are shown in Table 1. To achieve high range resolution and ensure that individual fish bodies possess sufficient pixel-level representation in the acoustic images, data acquisition was conducted entirely using the 1.2 MHz high-frequency mode. In this mode, the sonar has a horizontal beam angle of 80°, a vertical beam angle of 12°, a maximum effective detection range of 40 m, and 512 beams. Raw sonar data were stored in DAT format and decoded using the manufacturer’s Image Sonar Data Transfer Protocol (V1.3).

This study focused on largemouth bass reared in high-density intensive aquaculture facilities. The experimental dataset comprised 400 high-resolution sonar images acquired under representative operational conditions (target fish dimensions: mean length 15.0±0.5 cm, mean width 5.0±0.3 cm; sonar range R=2.0 m). Ground-truth bounding boxes were manually annotated by experienced aquaculture technicians using the standard LabelMe framework. To prevent data leakage from temporal autocorrelation between consecutive frames (10 fps), data splitting was executed strictly at the independent recording sequence level. The training set comprised 320 images (80%) sampled from six preliminary recording sessions. An independent test set of 80 images (20%) was collected from two separate, unseen video sessions.

### 2.2. Experimental Design

#### 2.2.1. Stocking Abundance Gradient Experimental Design

To systematically evaluate the impact of varying fish density on the performance of the target detection and correction models, a gradient density control experiment was designed. A custom-designed three-dimensional net cage that matches the geometry of the sonar’s sector-shaped scanning plane was deployed in a controlled laboratory recirculating tank. The net cage was constructed with a sector-shaped cross-section (radius R=2.0 m, horizontal sector angle θ=80°, bottom area $A_{s}=2.793\;m^{2}$) and an effective water depth of $H_{cage}=1.0\;m$, yielding a total water volume of $V_{cage}\approx 2.793\;m^{3}$. Since the sonar emits an acoustic beam with a narrow vertical opening angle of 12°, the insonified layer at a 2.0 m range possesses a vertical thickness of approximately 0.42 m (effective insonified sampling volume $V_{beam}\approx 0.78\;m^{3}$). Consequently, free-swimming fish dynamically distributed between in-beam and out-of-beam spaces across the 1.0 m water column. Five stocking abundance gradients (20, 40, 60, 80, and 100 juvenile largemouth bass) were stocked to evaluate model performance under controlled target crowding. Continuous acoustic video streams were acquired at the native sonar capture frame rate of 10 frames per second (fps, 100 ms inter-frame interval) for 10 min per trial; this high-rate video feed provided consecutive frame sequences for the cross-frame tracking and kinematic velocity filtering modules. Separately, to evaluate static abundance distributions without confounding temporal auto-correlation between adjacent frames, non-overlapping snapshot frames were sampled at 10-s intervals (60 independent sample frames per trial) for discrete statistical boxplot evaluations across three replicate trials.

#### 2.2.2. Commercial-Scale Aquaculture Pond Experiment

**Figure 1:**
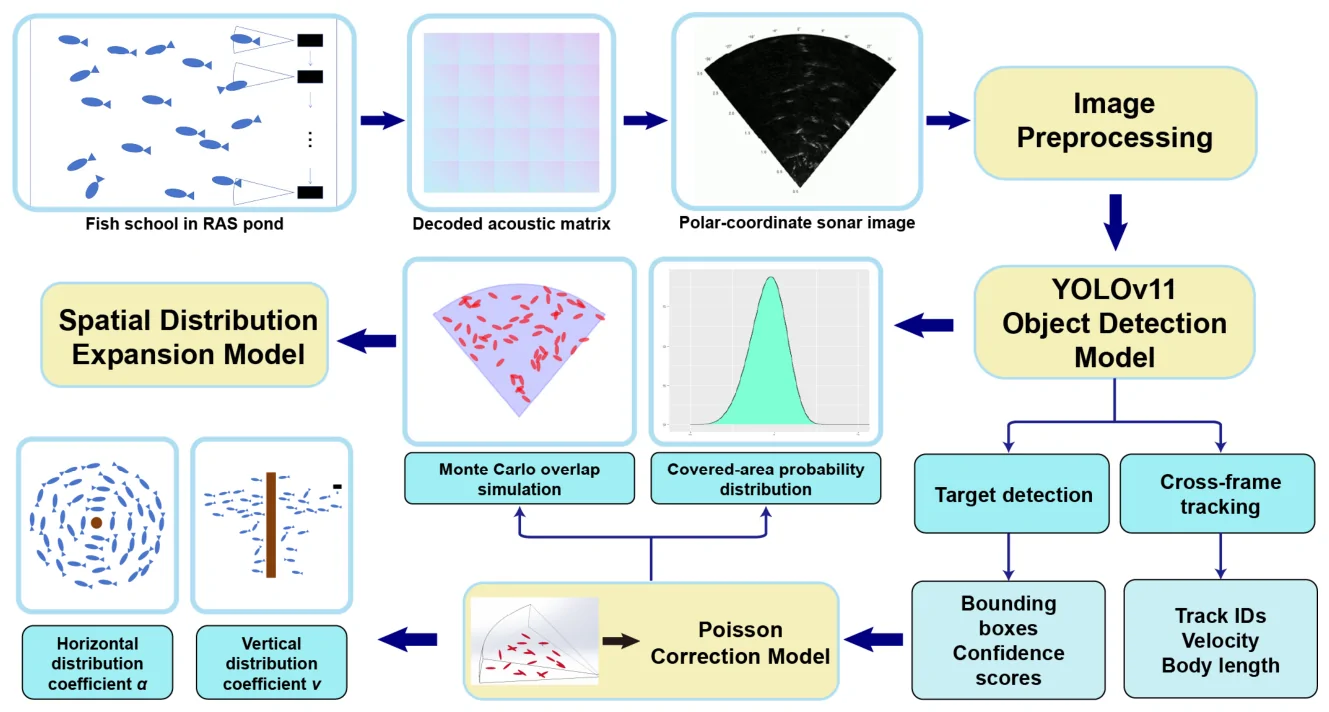
Overview of the end-to-end processing pipeline for fish stock estimation in intensive recirculating aquaculture systems (RASs). The tripartite framework encompasses sonar image preprocessing, deep-learning-based object detection and trajectory tracking (YOLOv11), statistical overlap correction via a Poisson coverage model, and whole-pond global abundance inversion governed by horizontal (α) and vertical (ν) spatial distribution coefficients.

Field validation experiments were conducted at the recirculating aquaculture facility (Hangzhou Hengze Ecological Agriculture Technology Co., Ltd., Hangzhou, China). The experimental design is illustrated in Figure 1. Two square concrete aquaculture ponds with horizontal dimensions of 7.5 m×7.5 m (bottom area $S_{pond}=56.25\;m^{2}$) and an operating water depth of H=2.0 m (total water volume $V_{total}=112.5\;m^{3}$) were designated as Pond A and Pond B. The target species was juvenile largemouth bass from the same production cohort, characterized by an average total body length of 15.2±1.1 cm and an individual wet body weight of 48.5±3.2 g. Reference baseline populations were established from initial batch stocking census records provided by the facility management, quantified as 6000±100 individuals for Pond A and 6500±100 individuals for Pond B (corresponding to an operational baseline uncertainty of approximately ±1.5%). The corresponding stocked biomass was approximately 291.0 kg and 315.3 kg, resulting in whole-pond mean stocking densities of $2.59\;kg/m^{3}$ ($53.3\;ind./m^{3}$) and $2.80\;kg/m^{3}$ ($57.8\;ind./m^{3}$), respectively. In industrial RAS nursery operations, social schooling creates pronounced spatial stratification; during daylight monitoring, fish strongly aggregate within the core 0.2–1.2 m depth layer, yielding local aggregation densities exceeding 12–18 kg/m^3^ (>250 ind./m^3^), which reflects representative commercial high-density conditions. Importantly, the acoustic validation trials were strategically executed immediately following the standardized nursery grading and batch-stocking census within a 24-h acclimation window. Because the interval was exceptionally brief, dynamic demographic variables common over extended breeding cycles—such as natural mortality, selective grading, or batch partitioning—were essentially absent. Daily facility mortality retrieval logs recorded zero fatalities during this window (<0.05%), confirming that the initial stocking records (6000±100 and 6500 ±100 individuals) provided a reliable operational reference baseline.

The imaging sonar transducer was mounted on an adjustable stainless steel vertical positioning rig at the pond boundary, with its central acoustic beam axis fixed strictly in a horizontal side-looking orientation (0° tilt angle). The horizontal wide-aperture beam (θ=80°) interrogated the horizontal plane, while the narrow aperture (ϕ=12°) restricted vertical elevation spread, yielding an instantaneous sector interrogation volume of $V_{beam}=\frac{1}{3}R^{3}\theta sin\phi \approx 0.78\;m^{3}$ at the maximum detection range R=2.0 m (horizontal sector area $A_{s}=2.793\;m^{2}$). To acquire vertical population profiles across the 2.0 m water column, a vertical stepping protocol was implemented across 11 discrete depth tiers (K=10 layer intervals). Starting from the subsurface (z=0.0 m), the transducer was translated downward in 0.2 m increments to the pond bottom (z=2.0 m), conducting stationary 10-s horizontal recordings at each tier. Because the transducer was translated vertically without tilt, the acoustic beam interrogated the target fish school from an identical horizontal side-view aspect across all stratified layers. Throughout data acquisition, dissolved oxygen (>6.5 mg/L), water temperature (24.5±0.5 °C), and pH (7.4±0.2) were rigorously maintained, and the fish were acclimated for 24 h prior to acoustic logging to avoid stress-induced anomalies.

#### 2.2.3. Offshore Net Cage Field Demonstration Setup

To evaluate the cross-species adaptability and engineering robustness of the framework in open-sea environments, field validation trials were conducted at a commercial deep-sea mariculture facility in Hainan, China. Four circular gravity net cages (diameter: 20 m, water depth: ~7 m) stocking commercial-size golden pompano (*Trachinotus ovatus*) were surveyed. The high-frequency imaging sonar was deployed vertically along the cage edge to perform stratified profile scanning across discrete depth intervals. Unlike fusiform teleosts, adult golden pompano exhibits a deep-bodied, laterally compressed oval morphology. Because the imaging sonar interrogates the fish from a horizontal side-looking perspective, the relevant acoustic echo highlight corresponds primarily to the strongly scattering vertebral and swim-bladder core structures rather than the soft fin margins. Consequently, the effective acoustic echo profile is modeled as an ellipse where the major axis corresponds to total length L and the effective acoustic minor axis b reflects the high-intensity scattering core (yielding an empirical acoustic aspect ratio of *L*/*b* ≈ 5.8–6.0, with mean projected acoustic footprint $A_{e}$ ranging from $156.3\;{cm}^{2}$ to $187.0\;{cm}^{2}$). The empirical lateral projected area ($A_{e}$, ranging from $156.3\;{cm}^{2}$ to $187.0\;{cm}^{2}$ across cages) was statistically calibrated directly from segmented in-beam bounding-box measurements rather than assuming a slender fusiform width ratio. In each cage, acoustic sampling was conducted at an extended operational range of R=5.0 m across 13 stratified depth tiers, recording 300 valid frames per cage for qualitative engineering evaluation.

### 2.3. Monte Carlo Stochastic Simulation Experiment

Monte Carlo stochastic simulation was executed to quantify target overlap effects and calibrate parameters for the Poisson-based coverage model. To address systematic counting underestimation caused by spatial crowding in single-frame acoustic images, this simulation modeled the stochastic geometric overlapping behavior of fish schools. Simulated fish bodies, represented as geometric ellipses conforming to the target species’ morphological dimensions, were randomly allocated within a simulated sector-shaped sonar detection area. The iterative generation of 1000 independent coverage scenarios across various density gradients established the statistical correspondence between individual abundance and the cumulative projected area. This probabilistic framework verified the mathematical consistency of the analytical inversion formula and enabled the evaluation of detection degradation in the absence of extreme-density empirical datasets.

### 2.4. Algorithm Design

A fish abundance estimation algorithm based on imaging sonar data was developed to accurately quantify fish population size in high-density recirculating aquaculture systems (RASs) by integrating computer vision and statistical models. The algorithm adopted a modular architecture to realize an end-to-end processing pipeline from localized sampling to global estimation. The overall logic was structured into four core stages: data acquisition and preprocessing, target detection and tracking, occlusion quantity correction, and spatial distribution expansion (Figure 1).

First, acoustic images of fish schools in the aquaculture pond were acquired using high-frequency imaging sonar. Preprocessing techniques such as median filtering, Gaussian low-pass filtering, and morphological operations were applied to suppress noise and enhance target features, providing high-quality inputs for subsequent visual analysis. Second, the YOLOv11 object detection model was implemented to perform real-time fish recognition and localization on the preprocessed acoustic images, outputting bounding boxes, confidence scores, and class information. Third, during the occlusion correction stage, a coverage probability model based on the Poisson distribution was introduced to address missed detections and double counting caused by target overlap and acoustic shadows. An analytical formula for correcting fish count estimates from the YOLOv11 detection results was derived by establishing the mathematical relationship between the projected area of fish bodies and the coverage probability within the sonar sector, thereby reducing systematic errors from spatial overlap. Finally, during the spatial distribution expansion stage, a method for deriving spatial distribution coefficients based on the “localized roaming” behavior of fish schools was developed to overcome the limited detection range of the imaging sonar. By defining the horizontal distribution coefficient α and vertical distribution coefficient ν, an estimation model was constructed to extrapolate biomass from the local sonar sampling area to the entire aquaculture pond, enabling the prediction of total numerical abundance in high-density aquaculture environments.

#### 2.4.1. Imaging Sonar Image Preprocessing Algorithm

First, the acquired raw imaging sonar data were unpacked and decoded into a two-dimensional matrix. This matrix was then transformed from the polar coordinate system (range-beam coordinates) to the Cartesian coordinate system for planar visual representation and object localization. Subsequently, median filtering was applied to the matrix. This nonlinear filter replaced the central pixel value with the median value of the pixels within a neighborhood, effectively eliminating typical salt-and-pepper noise and speckle noise in the sonar images. A Gaussian low-pass filter was then applied for further processing. The Gaussian filtering smoothed the image and effectively suppressed high-frequency noise, although it caused slight edge blurring. Subsequently, contrast adjustment was performed via dynamic range compression and stretching. This step suppressed the visual prominence of noise in homogeneous background regions while enhancing the feature contrast of target areas, thereby facilitating their clear separation from the background. Finally, morphological operations were applied to optimize target morphology. Using a 3 × 3 circular structuring element, a closing operation (dilation followed by erosion) was performed to fill holes and gaps within targets, followed by an opening operation (erosion followed by dilation) to smooth target contours and disconnect small bridges, thereby obtaining a more compact and coherent representation of the target regions.

Quantification of denoising performance across five standard statistical metrics provided the baseline for evaluating the image preprocessing workflow. Global distortion and quality preservation were measured through the Peak Signal-to-Noise Ratio (PSNR). Structural similarity between the preprocessed and raw acoustic images was captured via the Structural Similarity Index Measure (SSIM) [20]. Boundary detail retention during filtering operations was verified through the calculation of the Edge Preservation Index (EPI) [21]. Additionally, noise suppression within homogeneous background regions was characterized by variance reduction and noise level reduction percentages.

#### 2.4.2. Object Detection Algorithm

To establish the baseline target recognition module, the state-of-the-art open-source YOLOv11 object detection model (developed by Ultralytics) was implemented. Built upon an advanced feature extraction backbone integrating C3k2 (cross-stage partial network with customizable kernel sizes) and C2PSA (cross-stage partial with spatial-channel self-attention) modules, paired with an enhanced PAN-FPN feature pyramid neck, the network outputs target bounding box coordinates, confidence scores, and class predictions. Operational detection thresholds were set to a confidence score of 0.25 and an IoU threshold of 0.5 for Non-Maximum Suppression (NMS). Detection performance was evaluated on the independent 80-image test set using standard object detection metrics, including Precision, Recall, mean Average Precision (mAP@0.5), and F1-score, establishing the visual baseline before feeding target counts into the downstream statistical correction modules.

#### 2.4.3. Cross-Frame Object Tracking Mechanism

To track fish movement trajectories, an inter-frame target association algorithm integrating spatial proximity metrics and Intersection over Union (IoU) matching was implemented [22,23]. Positional proximity and geometric overlap of targets between adjacent frames were combined to mitigate tracking errors induced by rapid fish maneuvering, morphological changes, and brief acoustic occlusions. Bounding boxes in consecutive frames were defined by their top-left and bottom-right coordinates to establish the baseline coordinate matrices. For each paired combination of detection boxes across frames, a spatial proximity score was derived via normalized Euclidean distance between center points, while a geometric overlap score was simultaneously computed using standard IoU formulations. A comprehensive matching score $S_{ij}$ was subsequently established by applying equal linear weights of 50% to both metrics. Identity assignment was governed by a preset similarity threshold. Targets exceeding this threshold maintained their historical tracking identifiers, whereas anomalous or non-matching boxes were initialized as novel trajectories. Kinematic parameters were subsequently extracted from the verified identity sequences. Instantaneous velocity was calculated from the pixel displacement of target center points between consecutive frames, which was converted to physical velocity scaled by the preset sonar range parameter R, maximum frame dimensions, and frame rate f. Similarly, individual fish body length was estimated by scaling the horizontal pixel width of the bounding box against the identical range-dependent spatial calibration factor. Under the side-view monitoring perspective, horizontal orientation was assumed to validate the approximation of physical fish length via bounding box widths.

#### 2.4.4. Acoustic Artifact and Clutter Filtering Algorithm

In underwater imaging sonar surveillance, acoustic reverberation, tank boundary multipath reflections, structural fixtures (e.g., aeration pipes and net frames), and suspended bubbles frequently induce false-positive detections in deep learning object detectors. To ensure data purity before the statistical overlap correction stage, a spatiotemporal post-filtering module is integrated downstream of the YOLOv11 detection and cross-frame tracking pipeline. This module applies a dual-channel filtering logic based on kinematic and geometric constraints to discard non-fish acoustic clutter.

The filtering module processes continuous trajectory sequences from the tracking module, where each target k in frame t possesses attributes including bounding box coordinates $B_{t}^{k}=[x1_{t}^{k},y1_{t}^{k},x2_{t}^{k},y2_{t}^{k}]$, tracking ID, instantaneous velocity $v^{k}(t)$, and estimated acoustic length $l^{k}(t)$. Clutter rejection is executed via two parallel channels: a kinematic filtering channel and a geometric scale filtering channel.

In the kinematic filtering channel, the module identifies stationary acoustic clutter, such as persistent wall multipath reflections and static bottom reverberations, which exhibit near-zero spatial displacement across consecutive frames. The algorithm computes the global reference velocity $\bar{v}$ of all tracked active targets within a temporal observation window T:

(1) $$\bar{v}=\frac{1}{N_{T}}\sum_{t\in T}\sum_{k\in K_{t}}v^{k}\left(t\right)$$

where $N_{T}$ denotes the total number of velocity observations, and $K_{t}$ is the set of valid tracked targets in frame t. To prevent valid fish exhibiting brief resting pauses from being erroneously removed, a temporal persistence criterion is enforced. A detected bounding box is flagged as static background clutter only if its velocity consistently remains below the kinematic velocity rejection threshold over F consecutive frames (F = 10 in this study):

(2) $${Condition}_{V}:v^{k}\left(t\right)<\alpha_{v}\cdot \bar{v}$$

where $\alpha_{v}$ denotes the kinematic rejection coefficient (set to 0.5, ensuring targets whose velocities fall below 50% of the moving baseline $\bar{v}$ are flagged as static echoes). This threshold identifies both stationary wall reflections and drifting non-fish scatterers.

In parallel, the geometric scale filtering channel suppresses acoustic false positives generated by small bubble clusters, side-lobe speckle noise, or large structural pond boundaries whose dimensions deviate drastically from physical fish echo projections. The real-time baseline acoustic length ${\bar{l}}_{t}$ is evaluated across valid targets in the current sliding window:

(3) $${\bar{l}}_{t}=\frac{1}{\left|K_{t}\right|}\sum_{k\in K_{t}}l^{k}\left(t\right)$$

For each target k in the current frame, its estimated acoustic length $l^{k}(t)$ is evaluated against empirical morphological gating bounds:

(4) $${Condition}_{L}:l^{k}\left(t\right)>\beta_{upper}\cdot {\bar{l}}_{t}\;or\;l^{k}\left(t\right)<\beta_{lower}\cdot {\bar{l}}_{t}$$

where $\beta_{upper}$ and $\beta_{lower}$ are the upper and lower geometric gating thresholds, respectively. Bounding boxes that violate these physical bounds are discarded as acoustic speckle noise or structural fixtures, ensuring that only verified fish targets are supplied to the subsequent Poisson quantity correction stage.

#### 2.4.5. Occlusion-Induced Count Correction Algorithm

In high-density aquaculture environments, fish schools inherently display complex social schooling, rheotaxis, and vertical stratification governed by ambient hydrodynamic and dissolved oxygen gradients, thereby violating global Complete Spatial Randomness (CSR) across the whole water body. To resolve this spatial heterogeneity without compromising analytical tractability, our framework establishes a multi-scale spatial decoupling strategy. At the macro-scale, whole-pond spatial clustering, localized roaming boundaries, and vertical water-layer preferences are explicitly parameterized by the empirical horizontal roaming coefficient (α) and vertical distribution coefficient (ν) formulated in Section 2.4.6. Conversely, at the micro-scale, a single-frame imaging sonar ping interrogates an instantaneous, localized cross-sectional slice across a narrow sector (radial range R=2.0 m, vertical beam angle 12°, insonified volume $V_{beam}\approx 0.78\;m^{3}$). Within this confined instantaneous observation volume, the relative spatial coordinates and angular orientations of individual fish are dynamically decorrelated by high-frequency swimming maneuvers and local fluid turbulence. Consequently, a spatially homogeneous Poisson point process is adopted strictly as a localized first-order approximation to model the stochastic geometric overlapping and radial acoustic shadowing of projected fish ellipses on the two-dimensional image plane. This formulation isolates micro-scale visual detection saturation within the insonified slice, while delegating macroscopic schooling aggregation to subsequent volumetric and spatial-scale expansion.

The imaging sonar emits acoustic waves outward from the transducer vertex to form a sector-shaped detection area on the two-dimensional image plane. This region is geometrically parameterized by the maximum detection range R (in meters) and the horizontal beam angle θ (in degrees, where θ=80° in high-frequency mode).

Accordingly, the effective interrogation area of the sonar on the two-dimensional horizontal image plane is a sector with radius R and horizontal aperture angle θ, with area $A_{s}$ given by:

(5) $$A_{s}=\frac{\theta }{360^{\circ }}\cdot \pi R^{2}$$

For the high-frequency imaging mode with $\theta =80^{\circ }$, the sector area simplifies directly to $A_{s}=\frac{2\pi R^{2}}{9}$. This geometric relationship serves as the baseline for subsequent spatial statistical calculations.

In two-dimensional high-frequency imaging sonar, acoustic echoes are mapped onto a planar range–azimuth display, collapsing target elevation across the narrow 12° vertical aperture. Because cultured fish maintain a predominantly horizontal swimming posture in aquaculture tanks, the horizontal-looking sonar beam insonifies the school primarily from the lateral aspect. Consequently, the acoustic projection of a fusiform fish is approximated as an ellipse on the 2D image plane: the major axis length a corresponds to physical body length (snout to tail), while the minor axis length b corresponds to the effective acoustic body width.

Eccentricity: The eccentricity e of the ellipse is defined to describe the degree of elongation of its shape:

(6) $$e=\sqrt{1-\frac{b^{2}}{a^{2}}}$$

The aspect ratio and eccentricity e characterize the species-specific acoustic footprint. Juvenile largemouth bass display a characteristic fusiform morphology with an eccentricity of e≈0.94 (body length a≈0.15 m, width b≈0.05 m), generating well-defined elliptical echo highlights on the 2D acoustic plane. Because the sonar maintains an identical horizontal-looking attitude across all vertical stepping layers, the projected target cross-sectional area $A_{e}=\frac{\pi }{4}ab$ remains geometrically invariant across depth strata. Consequently, the average projected area $A_{e}$ of an individual fish in the sonar image is formulated as:

(7) $$A_{e}=\frac{\pi ab}{4}=\frac{\pi a^{2}\sqrt{1-e^{2}}}{4}$$

Assume there are n fish within the sector area. The total theoretical maximum coverage area of all fish is $nA_{e}$. Define the coverage density μ as the expected number of layers of coverage per unit area:

(8) $$\mu =\frac{nA_{e}}{A_{s}}$$

Substituting the expressions for $A_{s}$ and $A_{e}$ yields:

(9) $$\mu =\frac{9nab}{8R^{2}}$$

To provide a theoretical foundation for modeling visual detection saturation under acoustic shadowing and mutual overlap, the spatial distribution of target projections within the insonified 2D slice is conceptualized using the Boolean model of random closed sets. Let the projected centroids of fish targets within the sector region $A_{s}$ be modeled as a spatial Poisson point process with local intensity $\lambda =n_{slice}/A_{s}$ (the “germs”), with each fish generating an independent elliptical projection of average cross-sectional area $A_{e}=\frac{\pi }{4}ab$ (the primary “grains”). Under this stationary Boolean framework, the spatial coverage fraction $P_{cover}$, defined as the probability that an arbitrary coordinate on the sonar imaging plane is covered by at least one target echo projection, is analytically given by the complement of the void probability:

(10) $$P_{cover}=1-exp(-\mu )=1-exp(-\frac{nA_{e}}{A_{s}})$$

Under severe spatial crowding, the deep learning detector (YOLOv11) only successfully resolves targets occupying unoccluded front-layer positions. Consequently, the upper bound of visually resolvable targets within the sector region scales proportionally with the projected spatial coverage ratio $P_{cover}$. The expected number of detectable fish targets, denoted as $E\left[n_{yolo}\right]$, corresponds to the effective covered area normalized by the single-target projection area $A_{e}$:

(11) $$E\left[n_{yolo}\right]=\frac{A_{s}}{A_{e}}\cdot P_{cover}=\frac{A_{s}}{A_{e}}\left[1-exp(-\frac{nA_{e}}{A_{s}})\right]$$

Equation (11) mathematically establishes the nonlinear relationship between the true in-slice target abundance $n_{slice}$ and the expected visual detections $E\left[n_{yolo}\right]$ under occlusion constraints. Inverting Equation (11) to solve for the true fish count $n_{slice}$ as a function of the observed detection count $n_{yolo}$ yields:

(12) $$n_{slice}=-\frac{A_{s}}{A_{e}}\ln\left(1-\frac{{n_{yolo}A}_{e}}{A_{s}}\right)$$

Substituting the expressions for the sector area $A_{s}=\frac{2\pi R^{2}}{9}$ and the single-fish elliptical projected area $A_{e}=\frac{\pi ab}{4}$ into Equation (12), the closed-form formulation for rectifying planar counting saturation within the insonified 2D acoustic slice is obtained:

(13) $$n_{slice}=-\frac{8R^{2}}{9ab}\ln\left(1-\frac{9n_{yolo}ab}{8R^{2}}\right)$$

This closed-form formulation statistically rectifies the visual counting saturation caused by mutual fish overlap and acoustic shadowing within the insonified 2D detection slice. Mathematically, Equation (13) exhibits a singularity as the observed count approaches the theoretical packing saturation capacity, $n_{yolo}\to A_{s}/A_{e}\approx 474\;ind.$, where the logarithmic argument vanishes. In operational aquaculture environments, however, the maximum raw frame detection observed in our gradient experiments was 45.69 individuals, corresponding to an acoustic coverage saturation of only 9.64% ($u=n_{yolo}A_{e}/A_{s}\approx 0.096$). In commercial RAS ponds, single-frame detections typically average 13.4~18.9 individuals (u<4.0%). Real-world aquaculture operations are therefore strictly confined to the well-behaved, stable linear-to-moderate nonlinear regime (u ≤0.50), safely isolated from the mathematical asymptote. To prevent numerical divergence during embedded edge processing from spurious detector false positives, an operational guard clamp is enforced such that $u\leq \beta_{max}=0.95$.

To characterize how input uncertainties propagate to the in-slice corrected count, a first-order Taylor expansion (Delta method) is formulated:

(14) $$\sigma_{n_{slice}}^{2}\approx {\left(\frac{\partial n_{slice}}{\partial n_{yolo}}\right)}^{2}\sigma_{n_{yolo}}^{2}+{\left(\frac{\partial n_{slice}}{\partial A_{e}}\right)}^{2}\sigma_{A_{e}}^{2}$$

Evaluating the partial derivatives yields:

(15) $$\frac{\partial n_{slice}}{\partial n_{yolo}}=\frac{1}{1-\frac{n_{yolo}A_{e}}{A_{s}}}=\frac{1}{1-u},\frac{\partial n_{slice}}{\partial A_{e}}=\frac{A_{s}}{A_{e}^{2}}ln\left(1-u\right)+\frac{n_{yolo}}{A_{e}(1-u)}\approx \frac{n_{yolo}^{2}}{2A_{s}}$$

In an aquaculture enclosure ($V_{cage}\approx 2.793\;m^{3}$), fish roam freely across the entire water column while the sonar samples only a narrow horizontal cross-section ($V_{beam}\approx 0.78\;m^{3}$). Consequently, raw detection deficits originate from two distinct physical mechanisms: planar acoustic shadowing within the insonified 2D slice, and out-of-beam swimming coupled with boundary truncation. To extrapolate the in-slice corrected count $n_{slice}$ to the total enclosure abundance $N_{total}$, an effective volumetric calibration factor $\eta_{v}$ is cascaded downstream:

(16) $$N_{total}=\frac{n_{slice}}{\eta_{v}}=-\frac{8R^{2}}{9ab\eta_{v}}ln(1-\frac{9n_{yolo}ab}{8R^{2}})$$

where $\eta_{v}\in (0,\;1]$ represents the effective volumetric occupancy fraction interrogated by the acoustic beam. Under an idealized uniform spatial distribution, $\eta_{v}$ corresponds to the geometric volume ratio $V_{beam}/V_{cage}\approx 0.78/2.793\approx 0.279$. In intensive culture enclosures, however, fish display pronounced mid-water aggregation (as documented in Section 3.4), while acoustic sector boundaries induce edge-truncation detection losses. Consequently, following acoustic survey principles, $\eta_{v}$ operates as an empirical volumetric calibration parameter ($\eta_{v}=\int_{z\in beam}P\left(z\right)dz$) that absorbs behavioral vertical stratification and boundary baseline offsets, decoupling micro-scale planar occlusion from macro-scale 3D enclosure extrapolation.

#### 2.4.6. Whole-Pond Overall Estimation Algorithm

In high-density RASs, the behavioral patterns of target fish species (e.g., largemouth bass) exhibit distinct characteristics that differ from those in natural waters. Their spatial distribution is constrained by the physical boundaries of the aquaculture pond and the homogenized artificial environment, resulting in the loss of long-distance, directional periodic migration behavior. Instead, they display localized, high-frequency roaming in small areas around resource points such as feeding stations and aeration devices. This “localized roaming” behavior constitutes the foundation of their spatial distribution pattern. To quantitatively characterize this distribution pattern and thereby correct the observed density for estimating the true population size, this section proposes a two-level correction model incorporating a horizontal distribution coefficient and a vertical distribution coefficient.

The horizontal distribution coefficient α was defined to quantify the non-uniformity of horizontal spatial utilization caused by schooling tendencies, behavioral patterns, and environmental gradients. This coefficient represented the ratio of the typical fish activity area to the total bottom area of the aquaculture pond. The core parameter, the activity radius $R_{fish}$, represented the equivalent radius of the horizontal localized roaming area and was determined via statistical analysis of long-term trajectory data. Given the total horizontal pond area S, the coefficient was calculated as:

(17) $$\alpha =\frac{\pi R_{fish}^{2}}{S}$$

For the commercial experimental ponds ($S_{pond}=56.25\;m^{2}$), the effective horizontal roaming radius $R_{fish}$ was statistically calibrated from baseline planar trajectory centroid dispersion during non-feeding periods. The fish schools exhibited coordinated peripheral cruising with an equivalent localized gyration radius of $R_{fish}\approx 2.10\;m$, enclosing an active roaming patch area of $S_{roam}=\pi R_{fish}^{2}\approx 13.85\;m^{2}$. This yielded an empirical horizontal distribution coefficient of α=13.85/56.25≈0.246.

Values of α ranged within (0, 1], where a value approaching 1 indicated a wide, uniform distribution, and a smaller value signified a highly concentrated horizontal aggregation, analogous to the ecological ratio of home range area to total habitat area.

The vertical distribution coefficient ν was established to characterize vertical stratification driven by gradients of dissolved oxygen, temperature, light, and physiological states. The water column was evenly divided into K layers, and the instantaneous fish count $n_{i}$ within the i-th layer was estimated from the sonar profile data. The cumulative count sequence $C_{k}$ from the water surface to the k-th layer was calculated as:

(18) $$C_{k}=\sum_{i=1}^{k}n_{i}$$

The coefficient ν was defined as the proportion of the minimum number of consecutive layers containing 80% of the total population to the total layer count. The vertical coefficient ν was determined by identifying the minimum layer span (t−s+1) that satisfies $C_{s,t}/C_{K}\geq 0.8$, where $C_{s,t}$ represents the cumulative fish abundance from layer s to layer t (with s and t denoting the starting and terminal layer indices, respectively):

(19) $$\nu =\frac{t-s+1}{K}$$

Values of ν ranged within (0, 1], with 1 indicating uniform vertical distribution and lower values reflecting concentration within fewer water layers, drawing on the ecological concept of the population core distribution range.

Across the H=2.0 m water column divided into K = 10 equal-interval layers (11 sampling tiers at 0.2 m increments), vertical acoustic profiling demonstrated that the fish formed a prominent aggregation. The minimum consecutive water column enclosing the core population (0.2 m to 1.2 m) spanned 5 layer intervals (physical thickness 1.0 m). Consequently, the vertical distribution coefficient was determined as ν=0.50.

Methodologically, the stratified scanning protocol operates as a continuous vertical profiling procedure to parameterize the non-dimensional spatial coefficient ν. In Equation (21), overall abundance is estimated from the peak localized density (ρ) scaled by the effective roaming volume ($V_{occupied}$), rather than by accumulating detections across successive depth slices. Therefore, volumetric overlaps between adjacent acoustic beams introduce no mathematical double-counting into the global abundance inversion. Both α and ν were calibrated from independent exploratory pilot recordings, ensuring that spatial behavior parameters were decoupled from the validation trials.

During the stratified scanning process, the sonar sector detection volume V was assumed to align with and cover the core activity zone of the localized roaming fish school. Therefore, the fish count extracted from this localized space represented the peak local aggregation abundance within the pond. The parameter $n_{local}$ represents the actual fish abundance within the core roaming depth layer interrogated by the sonar scan. Because cultured fish in intensive RAS ponds exhibit strong vertical stratification (aggregating predominantly within the core stratum of thickness $∆z_{core}=H\cdot \nu \approx 1.0\;m$), a single horizontal acoustic slice (12° vertical aperture, physical thickness $∆z_{beam}\approx 0.42\;m$ at range R=2.0 m) interrogates a representative cross-sectional sub-volume. Consequently, the local aggregation abundance $n_{local}$ is recovered by cascading the micro-scale in-slice Poisson correction ($n_{slice}$, Equation (13)) with a vertical layer-occupancy ratio $\eta_{z}=∆z_{beam}/∆z_{core}\approx 0.569$:

(20) $$n_{local}=\frac{n_{slice}}{\eta_{z}}$$

Given that the fish school is concentrated within an effective roaming volume defined by the horizontal and vertical coefficients as $V_{occupied}=V_{total}\cdot \alpha \cdot \nu$, the peak core-layer fish density is expressed as $\rho =n_{local}/V_{beam}$. Extrapolating to the entire aquaculture pond, the total numerical abundance $n_{total}$ is derived by scaling the core density by the total occupied volume, yielding the spatial extrapolation formula:

(21) $$n_{total}=\rho \cdot V_{occupied}=\frac{n_{local}}{V_{beam}}\cdot V_{total}\cdot \alpha \cdot \nu =n_{local}\cdot S_{extrap}$$

where $S_{extrap}\approx 210.14$ represents the global volumetric extrapolation scaling factor calibrated for the commercial ponds.

## 3. Results

### 3.1. Acoustic Preprocessing and Object Detection Baseline

**Figure 2:**
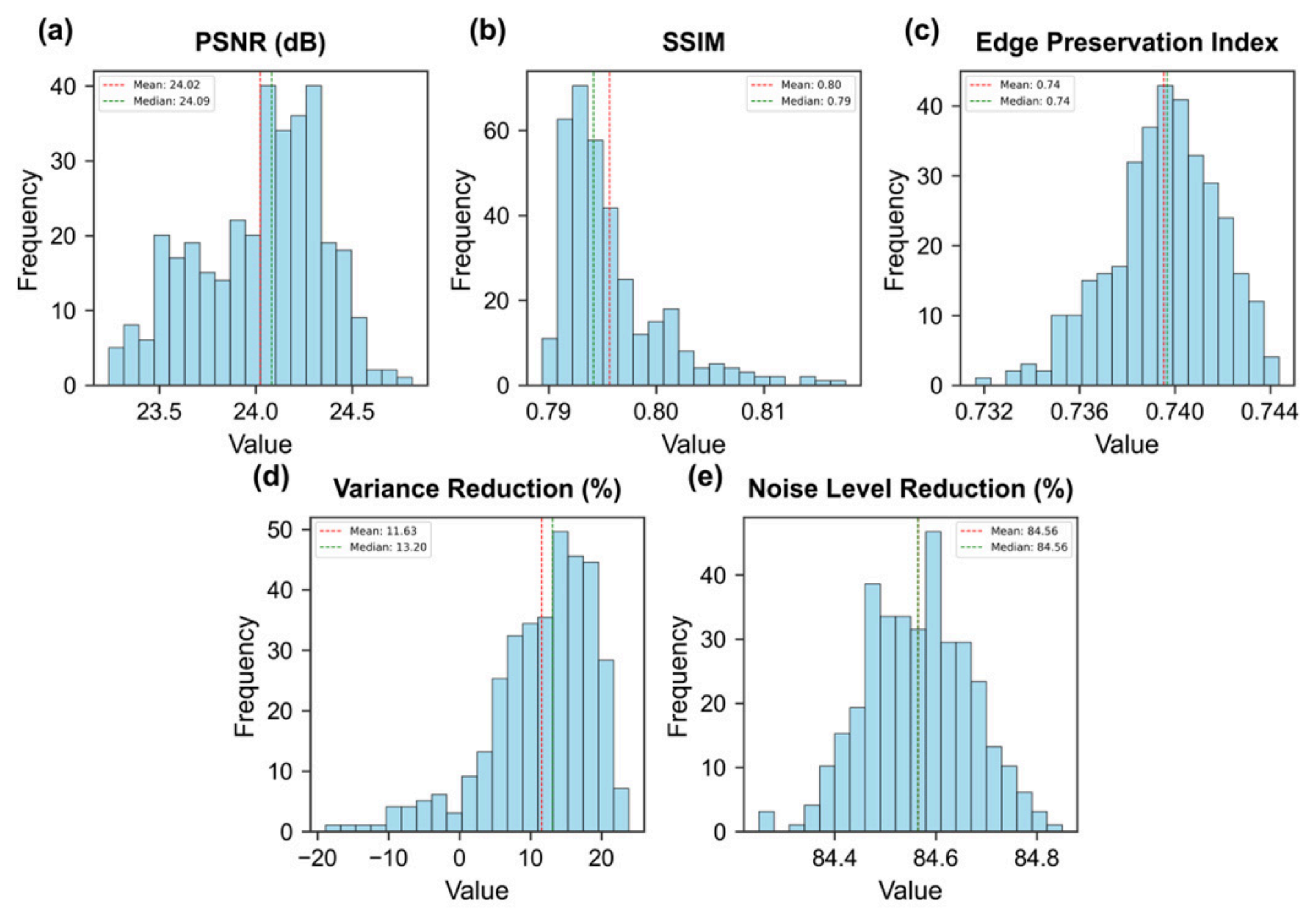
Statistical distributions of image quality and denoising metrics for sonar image preprocessing. Histograms display the frequency distributions of Peak Signal-to-Noise Ratio (PSNR), Structural Similarity Index Measure (SSIM), Edge Preservation Index (EPI), Variance Reduction (%), and Noise Level Reduction (%) evaluated across 346 acoustic data sets. Vertical red and green dashed lines represent the mean and median values, respectively.

A comparative analysis of denoised and non-denoised images was conducted using 346 sets of measured data (Figure 2). The PSNR values had a mean of 24.02, a median of 24.09, and a standard deviation of 0.35. The SSIM values had a mean of 0.80, a median of 0.79, and a standard deviation of 0.005. The EPI values had a mean of 0.74, a median of 0.74, and a standard deviation of 0.002. Variance reduction averaged 11.63% (median 13.20%, SD 7.63), and noise level reduction averaged 84.56% (median 84.56%, SD 0.10). These results indicate that the preprocessing workflow achieved effective noise suppression while preserving structural similarity and edge information. Evaluated on the unseen 80-image test set, YOLOv11 achieved a Precision of 89.1%, a Recall of 85.4%, an mAP@0.5 of 87.3%, and an F1-score of 0.872 at an inference latency of 6.8 ms/frame, confirming adequate acoustic discriminability and real-time operational capacity.

### 3.2. Monte Carlo Stochastic Simulation

**Figure 3:**
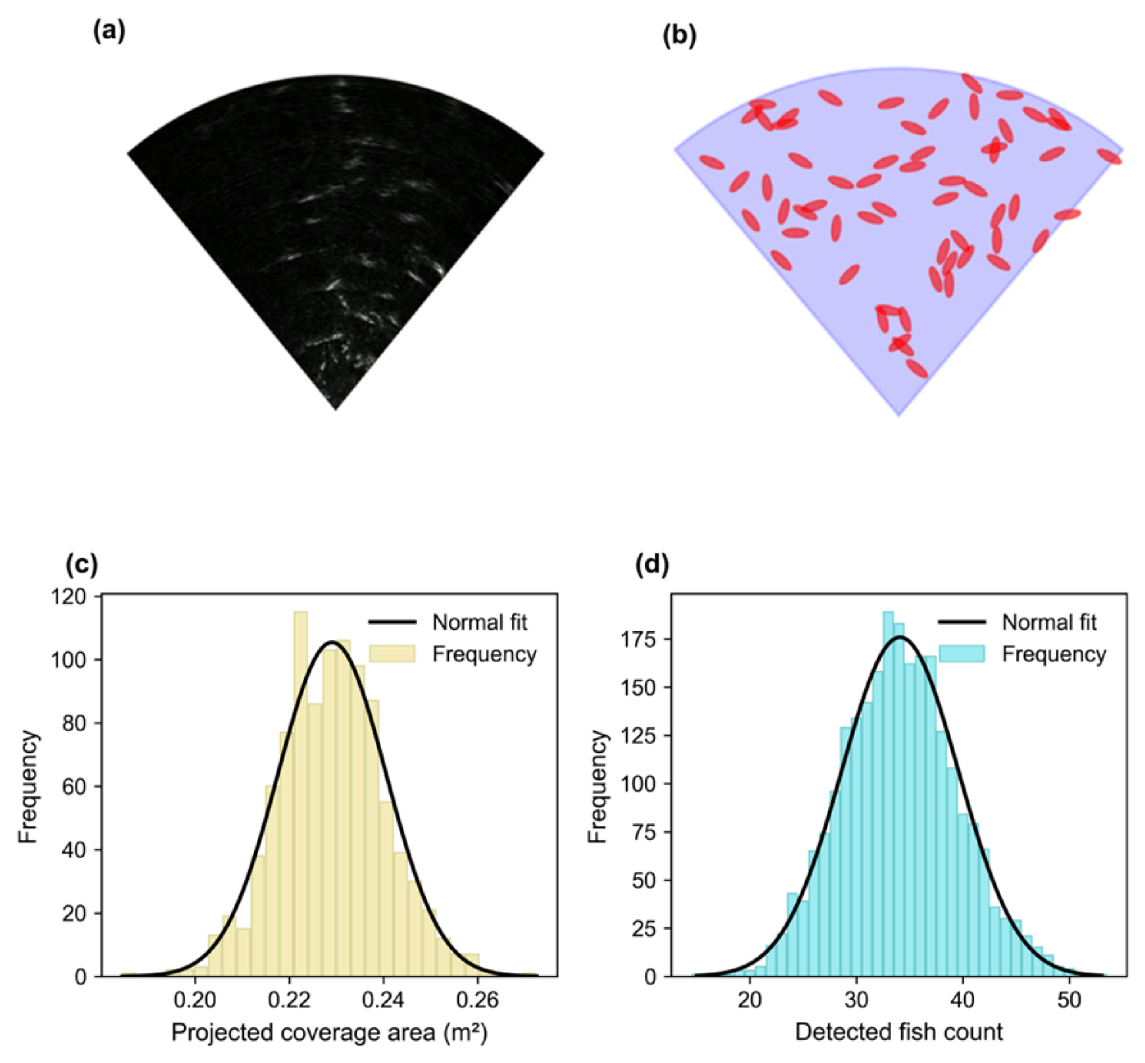
Comparison and probability distribution profiles between empirical sonar measurements and Monte Carlo stochastic simulations. (**a**) Decoded polar-coordinate imaging sonar sector; (**b**) simulated ellipse allocation model characterizing fish spatial distributions; (**c**) statistical frequency histogram and theoretical normal fitting curve for the projected coverage area (m^2^); (**d**) statistical frequency histogram and theoretical normal fitting curve for the detected fish count.

Figure 3 presents the systematic comparison between empirical sonar observations and Monte Carlo stochastic simulations across 1000 independent realization scenarios. In the simulation configuration, target bodies were modeled as planar ellipses (a=0.15 m,b=0.05 m) with uniformly distributed random angular orientations ($\theta \in \left[0,\;2\pi \right]$), and unconstrained spatial overlapping was permitted across the $2.793\;m^{2}$ sector across density gradients (*n* = 10–120). To evaluate distributional concordance between empirical observations and stochastic simulations, a two-sample Kolmogorov–Smirnov (K–S) goodness-of-fit test was conducted, yielding D = 0.048 (p = 0.62 > 0.05), confirming that the empirical observation distribution exhibits no statistically significant deviation from the theoretical stochastic realization. Furthermore, benchmark evaluations simulating a Thomas cluster point process (a stationary Neyman–Scott model with cluster dispersion radius σ=0.35 m) revealed that social schooling aggregation induces an average coverage deviation of only −3.03% (with maximum deviation bounded within −6.13% under n = 100) relative to the theoretical Poisson coverage curve. This verifies that the analytical Poisson formulation exhibits high stability across realistic biological clustering configurations.

### 3.3. Fitting Curves of the Poisson-Based Correction Model

**Figure 4:**
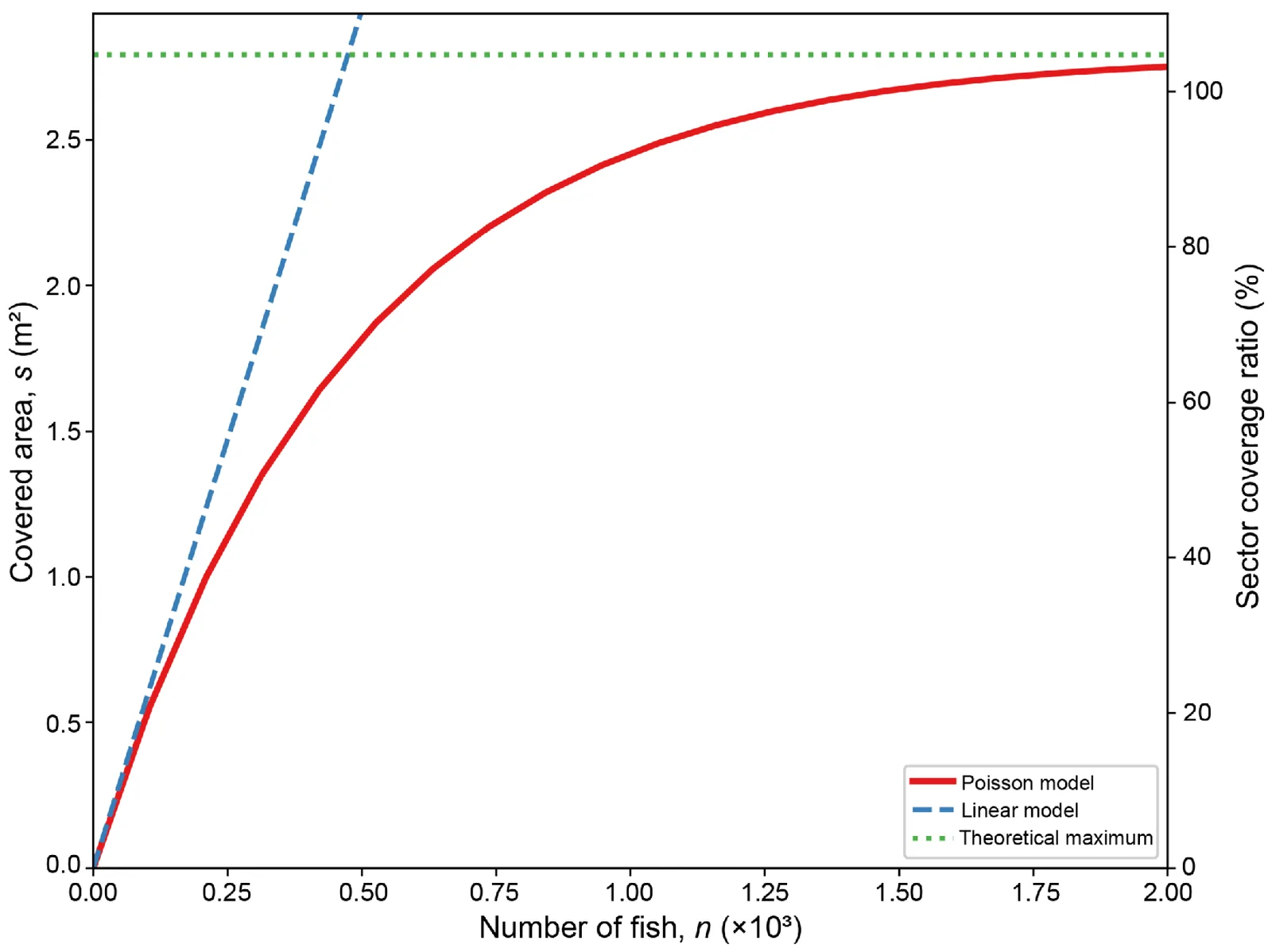
Comparison of Poisson-based and linear models for acoustic covered area (s) and coverage ratio versus fish abundance (n). Legend: Poisson model (red solid); linear model (blue dashed); theoretical maximum area of 2.79 m^2^ (green dotted). Parameters: fish size = 15 cm × 5 cm (area = 0.0059 m^2^), sonar range = 2.0 m, and beam angle = 80°. The 90% coverage thresholds are achieved at *n* = 426 (linear) and *n* = 1091 (Poisson).

A comparative analysis between the Poisson-based correction model and a linear model evaluated target overlap effects (Figure 4). The prediction curve of the Poisson-based model displayed asymptotic saturation. An initial rapid expansion of the coverage area slowed with the increase in fish population, asymptotically approaching the maximum sector area of 2.79 m^2^. Specifically, the linear model reached 90% sector coverage at a population of 426 fish, whereas the Poisson-based model required 1091 fish to achieve the same 90% coverage level. While the linear model exhibited unbounded growth beyond the physical sector limit, the Poisson-based model converged toward this theoretical upper bound.

### 3.4. Vertical Distribution of Fish

**Figure 5:**
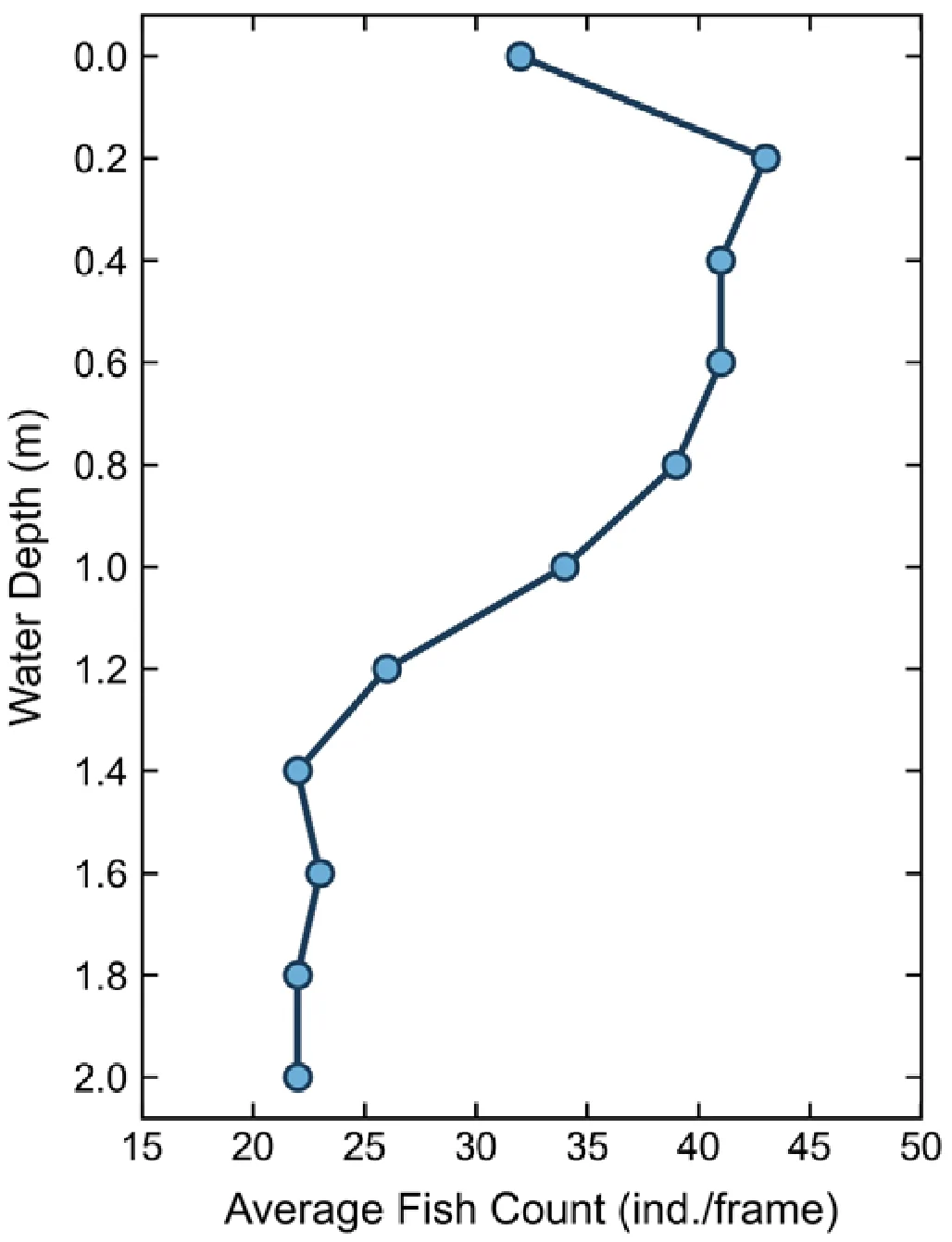
Continuous vertical distribution profile of fish across the water column in the intensive RAS pond. The horizontal axis represents the average fish count per acoustic frame (ind./frame) calculated across 346 validated acoustic image sequences. The vertical axis depicts the water depth (m) inverted from the water surface (0.0 m) to the pond bottom (2.0 m). Data markers represent frame-level spatial aggregation averages evaluated across 11 stratified vertical depth tiers ($z\in \left[0.0,\;0.2,\;0.4,\;\ldots ,\;2.0\;m\right]$ at 0.2 m increments).

Vertical acoustic profiling across 346 validated image sequences revealed marked population stratification: detection counts surged rapidly from the surface (0.0–0.2 m), formed a stable high-density aggregation plateau across the mid-upper column (0.2–1.0 m, peaking at 43 ind./frame at z=0.2 m), and decayed steadily toward the bottom. The core depth layer between 0.2 m and 1.0 m concentrated approximately 65% of the observed population, while extending the interval to 0.2–1.2 m (spanning 1.0 m thickness across 5 layer intervals) encompassed over 80% of the cumulative abundance ($C_{s,t}/C_{K}=80.6\%$), providing the empirical foundation for ν=0.50. Repeated vertical profiling across independent daily sequences confirmed that this stratified distribution remained highly stable, reflecting consistent pelagic habitat preference under standard RAS recirculating flow conditions (Figure 5).

### 3.5. Stocking Abundance Gradient Experimental Results

The detection characteristics of YOLOv11 and the recovery performance of the two-stage cascaded correction framework were systematically evaluated across five stocking gradients (Table 2). Raw YOLOv11 detections exhibited pronounced underestimation across all stocking levels, with relative errors ranging from 39.20% to 61.10%. Notably, the relative counting deficit was most severe at the lowest density (20 individuals, 61.10% error), where target overlap is theoretically negligible (<1%). This confirms that the severe counting deficit at low density is primarily driven by geometric out-of-beam swimming across the 1.0 m deep net cage rather than mutual acoustic shadowing. Conversely, at higher densities (80–100 individuals), mutual acoustic shadowing and target occlusion within the insonified layer progressively intensified as the population crowded into the core swimming zone.

**Figure 6:**
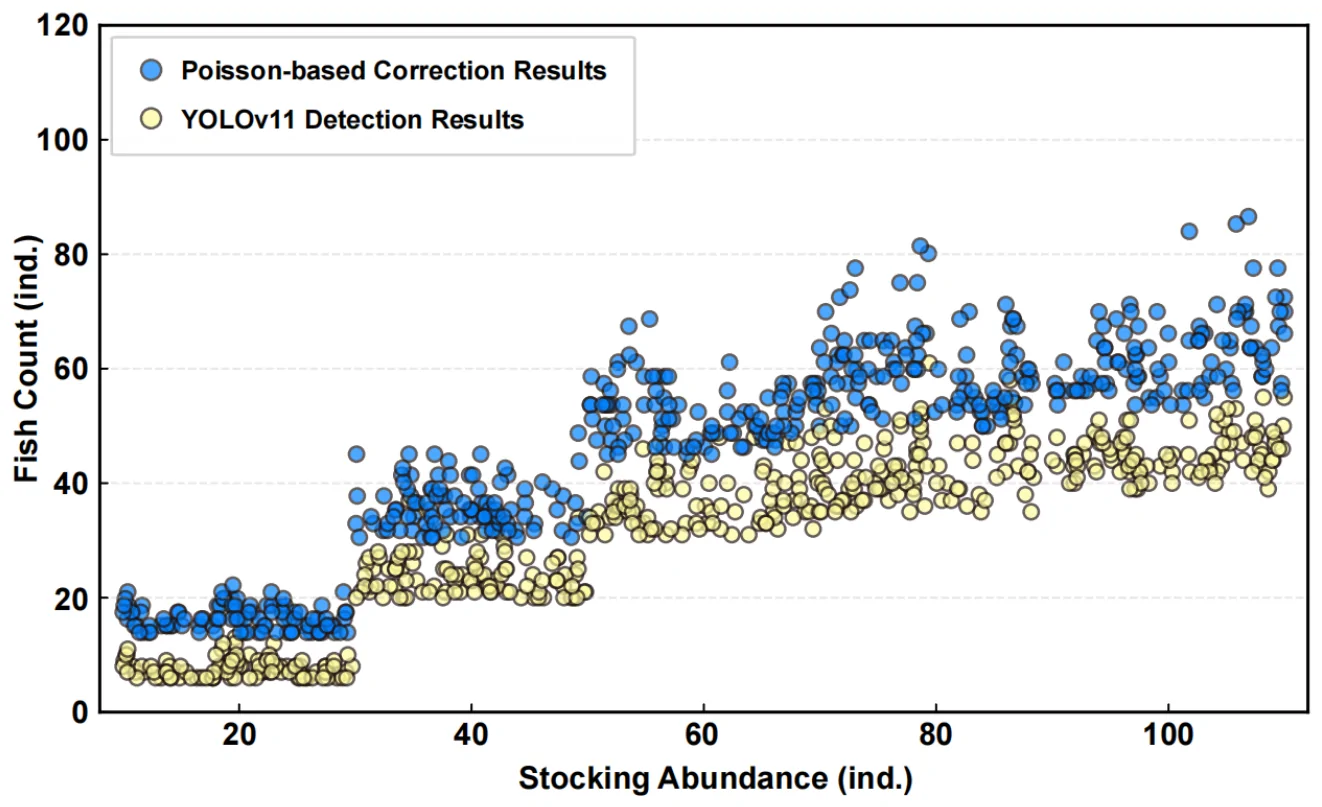
Fish count estimation under varying stocking abundance gradients: a frame-level comparison between raw YOLOv11 detection results (light yellow dots) and cascaded correction results combining micro-scale Poisson rectification with vertical occupancy expansion (blue dots). Controlled stocking levels range from 20 to 100 individuals. Abbreviation: ind., individuals.

To elucidate the computational pipeline, the 20-fish gradient serves as a representative worked numerical example evaluated at the frame level. The preset sonar parameters (h=2.0 m, θ=80°) and fish dimensions (a=0.15 m, b=0.05 m) establish an insonified sector area of $A_{s}=2.793\;m^{2}$ and single-target projected area of $A_{e}=0.00589{\;m}^{2}$, yielding a nominal saturation capacity ratio of $A_{s}/A_{e}\approx 474$. In Stage 1 (micro-scale slice correction), the algorithm processes continuous video sequences frame-by-frame: for a representative sample frame with raw detection $n_{yolo}=8$ ind., evaluating the planar Boolean-Poisson model (Equation (13)) yields an in-slice count of $n_{slice}=-474.07ln\left(1-8/474.07\right)\approx 8.07\;ind.$, which rectifies a localized mutual overlap loss of 0.07 individuals. In Stage 2 (volumetric enclosure expansion), accounting for net cage dimensions ($V_{cage}\approx 2.793{\;m}^{3}$, $V_{beam}\approx 0.78{\;m}^{3}$) alongside boundary truncation effects, the effective vertical occupancy under this sparse baseline evaluates to $\eta_{v}\approx 0.475$. Applying the volumetric calibration in Equation (16) yields an individual frame estimate of $N_{total}=8.07/0.475\approx 17.0\;ind.$ (visible as individual blue scatter points in Figure 6). Repeating this two-stage transformation across all 60 snapshot frames in the test sequence and calculating the arithmetic mean yields the final cascaded estimate of 16.50 individuals reported in Table 2, reducing the relative estimation error from 61.10% to 17.50%.

Applying this cascaded framework across all gradients, the in-slice Poisson formulation (Equation (13)) yields corrected slice counts $n_{slice}$ of 7.85, 24.79, 37.96, 44.71, and 48.04 individuals, confirming that planar acoustic occlusion increases nonlinearly from 0.065 to 2.354 individuals as target crowding intensifies. Concurrently, the effective vertical occupancy within the acoustic beam systematically shifts across densities ($\eta_{v}=0.475,\;0.685,\;0.732,\;0.751$, and 0.765 for 20 to 100 fish, respectively). This progression reflects a density-dependent behavioral transition: at low stocking densities, fish roam broadly across the unsonified depth boundaries, whereas elevated stocking density triggers tight social schooling that compresses the majority of the population into the central pelagic layer interrogated by the sonar beam. As a result, the ratio of restored total abundance to raw detections ($N_{total}/n_{yolo}$) exhibits the observed monotonically decreasing trend of 2.12, 1.50, 1.42, 1.40, and 1.37. As illustrated in Figure 6, post-correction relative errors ranged from 9.58% to 37.22%, with error reduction efficiency peaking at 75.84% under medium density (40 individuals) and maintaining 31.47% under extreme crowding (100 individuals).

### 3.6. Aquaculture Pond Experimental Results

**Figure 7:**
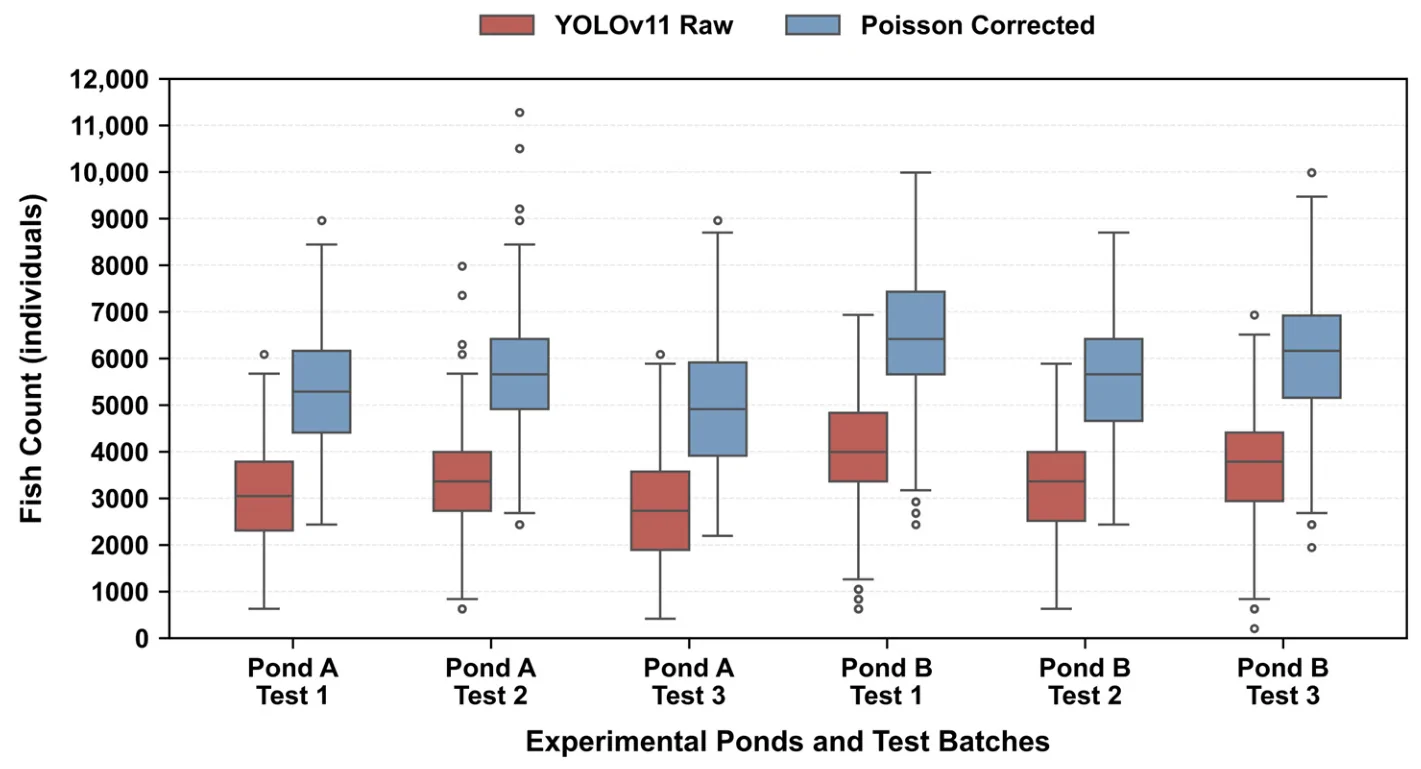
Frame-level fish abundance estimations from raw YOLOv11 detection versus Poisson correction across three replicate trials in RAS ponds A and B. Boxes represent the 25–75th percentiles (center line: median); whiskers indicate 1.5× IQR; circles denote outliers. Red-brown and blue boxes represent raw and corrected counts, respectively.

Validation results for Pond A (6000±100 fish) and Pond B (6500±100 fish) across three independent operational trials are summarized in Table 3 and Figure 7. To isolate the specific gain of the micro-scale occlusion correction from the macro-scale spatial extrapolation, Table 3 reports both the uncorrected whole-pond baseline ($N_{raw}$, obtained by directly extrapolating raw YOLOv11 detections using the spatial volumetric scaling factor without occlusion rectification) and the post-correction estimate ($N_{corr}$). Across all trials, raw YOLOv11 detections suffered severe underestimation, with whole-pond baseline relative errors ranging from 38.77% to 53.12% (mean 46.76%). For Pond A, uncorrected relative errors were 53.12%, 44.22%, and 49.43%; for Pond B, they were 38.77%, 44.80%, and 50.23%. Following micro-scale Poisson occlusion correction, estimation errors were substantially reduced to 1.38–16.43% (mean 9.91%), achieving an error reduction efficiency of 69.07–96.44%. The observed trial-to-trial variance in corrected estimates (e.g., relative errors ranging from 1.38% to 15.14% in Pond B) is primarily attributed to hydrodynamic circulation and localized fish aggregation around aeration inlets across single scan sequences, which can be mitigated in production via multi-frame temporal moving averages.

To illustrate the computational workflow, Pond A (Trial 1) serves as a representative worked numerical example demonstrating the step-by-step transition from raw frame detections to the whole-pond population estimate. Acoustic sampling across K=10 vertical layers (11 discrete depth tiers at 0.2 m increments) yielded an average raw detection count of $n_{yolo}=13.39\pm 5.21\;ind./frame$ across 402 valid sonar frames.

The abundance inversion executes through a three-stage cascade:

Stage 1 (micro-scale slice occlusion correction): Evaluating the closed-form Poisson coverage model (Equation (13)) with parameters $A_{s}=2.793\;m^{2}$ and $A_{e}=0.00589{\;m}^{2}$ ($A_{s}/A_{e}\approx 474.07$) rectifies planar target overlapping and acoustic shadowing within the 2D slice:

$$n_{slice}=-474.07ln\left(1-\frac{13.39}{474.07}\right)\approx 13.58\;ind./frame$$

which compensates for a mutual occlusion deficit of 0.19 ind./frame.

Stage 2 (vertical beam layer-occupancy recovery): Accounting for the vertical beam thickness (0.42 m) relative to the core aggregation depth span (0.74 m within the 0.2–1.0 m depth stratum), the vertical layer-occupancy factor evaluates to $\eta_{z}\approx 0.569$. Applying Equation (20) recovers the localized core-layer abundance:

$$n_{local}=\frac{n_{slice}}{\eta_{z}}=\frac{13.58}{0.569}\approx 23.86\;ind./frame$$

Stage 3 (macro-scale spatial extrapolation): Applying the calibrated whole-pond spatial extrapolation factor ($S_{extrap}\approx 210.14$, Equation (21)) yields the post-correction whole-pond abundance:

$$N_{corr}=n_{local}\times S_{extrap}=23.86\times 210.14\approx 5014\;ind.$$

For rigorous comparison, directly extrapolating the raw visual detections prior to occlusion and layer correction yields the uncorrected baseline:

$$N_{raw}=n_{yolo}\times S_{extrap}=13.39\times 210.14\approx 2813\;ind.$$

Because both $N_{raw}$ and $N_{corr}$ share the identical spatial extrapolation baseline ($S_{extrap}=210.14$), the marked error reduction from 53.12% to 16.43% (efficiency 69.07%) demonstrates that coupling localized acoustic calibration ($n_{slice}$ and $\eta_{z}$) with spatial roaming expansion effectively eliminates systematic counting underestimation in commercial high-density aquaculture.

### 3.7. Qualitative Engineering Demonstration and Cross-Species Adaptability in Commercial Offshore Net Cages

To assess the cross-species generalization and operational robustness of the acoustic–statistical framework in large-scale open-sea mariculture, field application trials were conducted across four commercial offshore deep-sea net cages stocking golden pompano. Due to the commercial stocking scale (tens of thousands of fish per cage) and the severe risk of stress-induced mortality, lethal full-cage harvesting for exact individual counting is unfeasible in operational open-sea aquaculture. Consequently, the commercial batch-stocking records provided by the facility management (60,000–70,000 individuals for standard-density cages and 80,000–90,000 individuals for the high-density cage) served as the macroscopic operational baseline for qualitative consistency evaluation.

**Figure 8:**
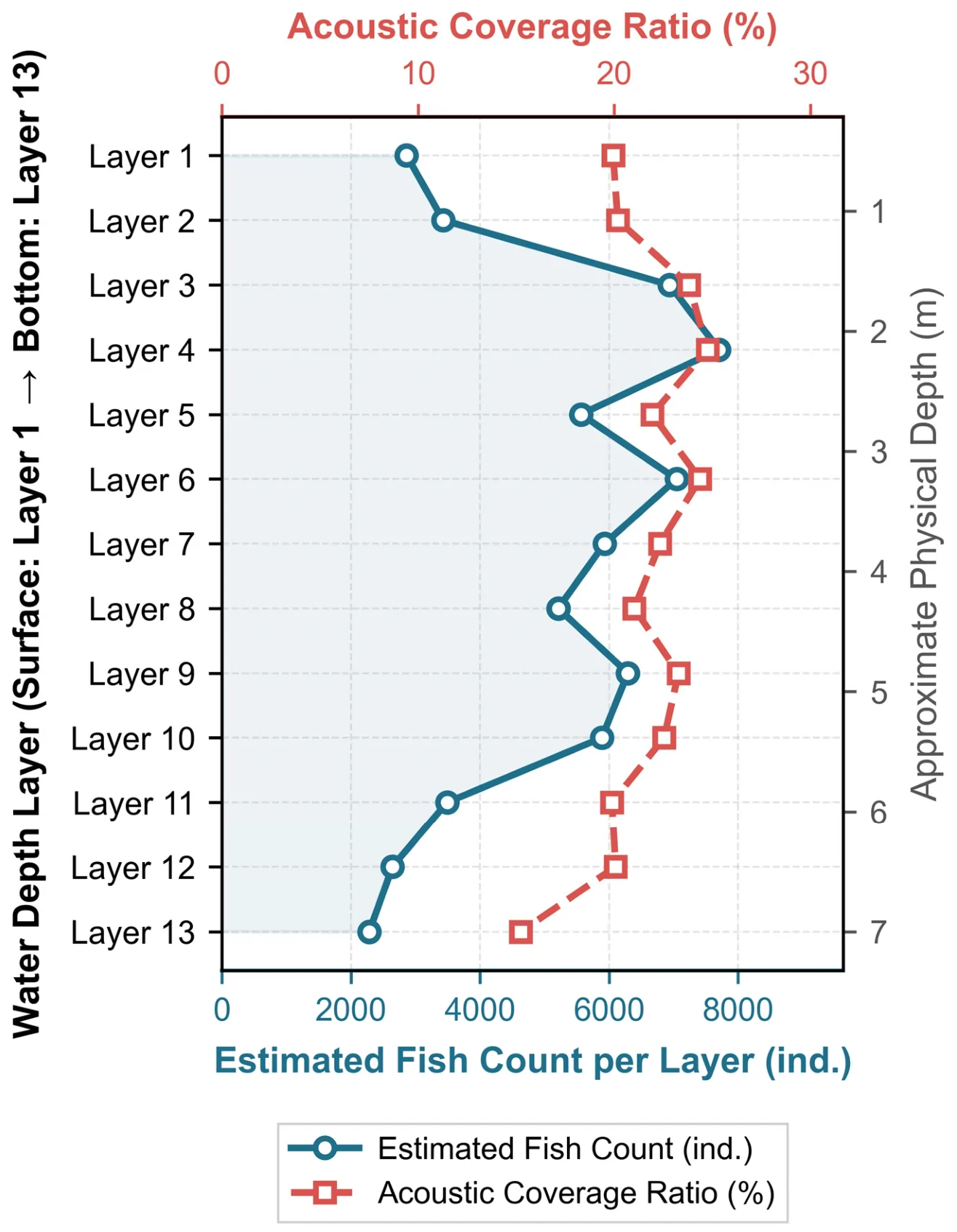
Continuous vertical distribution profile of golden pompano (*Trachinotus ovatus*) across 13 stratified water layers in commercial offshore deep-sea Cage 1. The profile demonstrates pronounced pelagic schooling aggregation in the intermediate water column (Layers 3–6), validating the framework’s vertical profile sensing capability in open-sea hydrodynamic environments.

The qualitative estimation results across all four commercial gravity cages are summarized in Table 4. Here, the mean acoustic surface coverage (%) is defined as the average percentage of pixels within the sonar interrogation sector occupied by segmented fish echo targets across valid frames. By incorporating the empirical lateral projected area ($A_{e}$) into the Poisson correction formulation, the framework adapted to the laterally compressed body profile of adult golden pompano without structural retuning of the detection head. For spatial extrapolation across the circular net cage (D=20 m,H≈7 m, volume $V_{cage}\approx 2200{\;m}^{3}$), spatial parameters were empirically configured as $\alpha_{cage}\approx 0.60$ (reflecting circular peripheral schooling) and $\nu_{cage}\approx 0.46$ (representing core pelagic occupancy across 6 layers in Figure 8).

The sonar-derived estimates exhibited consistent monotonic agreement with the macro-scale farm stocking tiers, though environmental disturbances induced modest numerical deviations. Cage 4, which exhibited the highest acoustic surface coverage (32.20%) and reduced swimming velocity (8.14 px/s), yielded an estimated abundance of 98,220 individuals. While this exceeds the nominal upper baseline (80,000–90,000) by approximately 9.1%, it plausibly captures severe localized crowding and net volume deformation driven by tidal currents. Conversely, Cages 1–3 exhibited lower acoustic coverage ratios (21.49–24.74%) with estimated abundances ranging from 56,794 to 65,269 individuals. The slight under-estimation in Cage 3 (~5.3% below the lower baseline) is reasonably attributed to cumulative unrecorded operational mortalities and target dispersion toward net boundaries. Overall, this open-sea trial substantiates the engineering viability and cross-species adaptability of the acoustic framework under challenging marine hydrodynamics, rather than claiming census-grade precision.

Furthermore, the 13-layer stratified scan in Cage 1 captured the continuous vertical distribution profile of the pelagic school in turbulent sea conditions (Figure 8). Golden pompano exhibited a distinct preferred swimming zone, with peak aggregation occurring within intermediate depth layers (Layers 3–6), while surface and net-bottom layers showed marked density reductions. This preliminary open-sea trial demonstrates that the integrated framework exhibits promising engineering viability for multi-layer spatial profiling and macroscopic density-tier tracking, showing potential for cross-species extension to marine cage aquaculture.

## 4. Discussion

### 4.1. Methodological Advantages and Performance

The integration of computer vision with spatial statistical theory in a tripartite framework of detection, correction, and expansion addresses the inherent limitations of underwater acoustic monitoring in intensive aquaculture, where challenges such as dense school identification and high data processing overhead often persist [1]. The sequential image preprocessing workflow, which combines median and Gaussian filtering with dynamic range adjustments and morphological operations, suppresses speckle noise and acoustic shadows while preserving target boundary definition. While certain motion-enhanced hybrid preprocessing pipelines can introduce information bottlenecks in high-capacity networks [24], our workflow establishes a high-quality baseline optimized for YOLOv11 object localization. This builds upon the broader successful application of standard and lightweight deep learning architectures for rapid underwater fish recognition and tracking across various water bodies [25,26,27].

To resolve the systematic counting underestimation caused by fish school occlusion, the introduction of a Poisson coverage model provides a mathematical link between observed bounding box projection areas and true target abundance. This aligns with recent advancements in handling severe target overlapping and body adhesion through density modeling and calibration mechanisms [28]. This visual and statistical synthesis establishes a robust error control mechanism, demonstrating the adaptability of spatial point processes in rectifying visual detection saturation within high-density environments, offering a reliable alternative or complement to sophisticated probabilistic Bayesian inversion methodologies used for multi-frequency density and uncertainty quantification.

A central theoretical inquiry regarding the micro-scale model concerns the validity of the spatial Poisson assumption given the social aggregation tendencies of cultured fish. To rigorously quantify the sensitivity of the closed-form correction to non-random spatial clustering, a synthetic Monte Carlo sensitivity analysis was conducted benchmarking the baseline homogeneous Poisson process against a Thomas cluster point process (a classic stationary Neyman–Scott model). Parent cluster centers were generated with spatial intensity κ, around which individual fish coordinates were distributed following an isotropic bivariate Gaussian dispersion kernel with standard deviation σ. The simulation evaluated two distinct clustering regimes across abundance levels (*n* = 20–100): compact crowding (σ=0.15 m) and typical swimming dispersion (σ=0.35 m, consistent with empirical nearest-neighbor distances in our sector). The results revealed that under typical operational schooling dispersions (σ=0.35 m), the mean projected coverage area deviates from the theoretical Poisson formulation by an average of only 3.03% (with maximum deviation bounded within 6.13% at n = 100). While severe compact clustering (σ=0.15 m) intensifies local overlap and reduces overall coverage by up to 25.77%, its primary effect is inflating single-frame variance rather than invalidating the central expectation. From an engineering perspective, inverting a non-stationary Neyman–Scott cluster model in operational edge-computing environments is mathematically ill-posed, requiring real-time estimation of latent cluster hyperparameters (κ,σ) that introduce severe numerical instability. In contrast, the closed-form Poisson formulation provides a deterministic, $O\left(1\right)$ computationally lightweight first-order estimator, maintaining robust bounded error while supporting high frame-rate edge processing.

Analysis of the stocking abundance gradient experiments reveals that raw YOLOv11 detection underestimation follows a pronounced U-shaped trajectory across densities (61.10% error at 20 fish, dipping to approximately 39% at intermediate densities, and reincreasing to 54.31% at 100 fish). This non-monotonic trend originates from the shifting dominance of two distinct physical error regimes rather than target occlusion alone. In sparse conditions (20 fish), physical target overlap within the acoustic slice is negligible (<1%), and detection deficits are predominantly governed by macroscopic geometric losses: free-swimming individuals dynamically disperse across the 1.0 m water column outside the narrow 12° vertical acoustic beam ($V_{beam}/V_{cage}\approx 0.279$). Under such conditions, volumetric expansion ($\eta_{v}\approx 0.475$) provides the primary corrective recovery. Conversely, as stocking abundance intensifies, crowding and social schooling compress the population into the central pelagic layer, increasing effective beam occupancy ($\eta_{v}\to 0.765$) while planar acoustic shadowing and mutual target occlusion surge nonlinearly as the dominant bottleneck. The two-stage cascaded architecture successfully decouples these scale-dependent processes, rectifying micro-scale 2D saturation via the analytical Poisson model while absorbing macro-scale behavioral dispersion through volumetric calibration. In traditional acoustic transect surveys, inaccurate geometric modeling of the insonified volume frequently induces substantial density and abundance estimation biases [29]. Furthermore, traditional acoustic quantification methods, such as volume backscattering ($S_{v}$) or target strength (TS) analysis, exhibit substantial calibration errors in dense aggregations [30,31]. These techniques depend heavily on empirical intensity-length relationships that are sensitive to fish species, body orientation, and environmental factors such as water temperature and depth.

In dense aquaculture monitoring, established computer vision methodologies generally follow two competing paradigms: end-to-end density-map regression (e.g., crowd-counting networks and FCFormer [12,13]) and bounding-box object detection. While density-map regression bypasses individual target bounding boxes by mapping acoustic feature representations directly to continuous density distributions, it operates essentially as an unconstrained black box. In real-world operational environments, sudden acoustic backscatter anomalies—such as aeration bubble bursts, feed pellet drift, and multi-path boundary reverberations—can severely distort feature embeddings, causing density regressors to suffer catastrophic drift without physical explainability. Conversely, bounding-box detectors paired solely with kinematic trackers neglect physical target occlusion, suffering acute saturation under severe spatial crowding.

Furthermore, existing sonar-based mariculture frameworks [17,18,19] primarily focus on in-beam localized target recognition, lacking mathematical mechanisms to compensate for volumetric sampling mismatches across the broader water column. The hybrid framework proposed herein circumvents these shortcomings by coupling high-speed discriminative detection with a transparent, physically grounded statistical layer. By inserting the closed-form Poisson model between the detector and the final count, mutual occlusion is rectified through explicit geometric grain parameters ($A_{e},A_{s}$), while spatial coefficients (α, ν) account for out-of-beam roaming. This modular architecture retains the spatial interpretability of individual detections while providing robust resilience against acoustic environment variations.

The practical application of this non-invasive acoustic monitoring framework provides a practical sensing approach for high-density recirculating aquaculture systems, matching or complementing the high frame rates required for resource-limited embedded deployments [32]. Continuous and accurate abundance quantification facilitates the optimization of daily feed intake, thereby improving feed conversion ratios, reducing production costs, and minimizing water quality degradation from feed waste, consistent with the identified goals of modern quantitative behavioral analytics [33]. Furthermore, integrating kinematic and morphological filtering enables automated exclusion of acoustic artifacts, stationary multi-path reflections, and non-target clutter before statistical quantity correction. Overall, the proposed sensing workflow demonstrates the feasibility of combining high-frequency imaging sonar with statistical spatial modeling to support automated population tracking in intensive aquaculture systems.

### 4.2. Driving Mechanisms of Vertical Stratification and Long-Term Model Robustness

The pronounced vertical stratification of largemouth bass within the subsurface water layer reflects a behavioral adaptation. This behavior is driven by a combination of hydrodynamic and illumination gradients inherent to intensive recirculating aquaculture systems. Comprehensive reviews indicate that tank hydrodynamics closely correlate with fish growth, welfare, and spatial distribution [34]. Although dissolved oxygen levels remain relatively homogeneous due to continuous system aeration, bottom flow dynamics act as the primary determinant of spatial allocation. The typical center-drain configuration generates elevated water velocities and shear stress near the pond floor to maximize solid waste removal. These hydrodynamics and structural self-cleaning efficiencies are highly sensitive to inlet piping configurations and flow velocities [35,36,37].

To minimize metabolic costs associated with position maintenance, the fish school preferentially occupies the upper intermediate pelagic zone, utilizing it as a hydraulic refuge. Numerical coupling models confirm that the physical presence and swimming of fish schools actively alter local flow fields, reducing water velocities and changing the structural uniformity of the pool [38]. This hydrodynamic preference is further reinforced by indoor light attenuation profiles, which establish a preferred illumination zone that aligns with the visual capabilities of the species. Thus, the vertical aggregation represents an optimization strategy balancing kinetic energy conservation with environmental preference. This hydrodynamically driven spatial stability is particularly relevant for the temporal reliability of acoustic profiling. Because data acquisition at each depth tier required only 10 s of stationary recording (completing full-depth profiling within several minutes) and surveys were conducted during non-feeding resting periods, juvenile largemouth bass maintained a quasi-stationary vertical distribution. The fish exhibited an affinity for low water velocities in the mid-upper column, exhibiting negligible rapid vertical migration during the short sampling window.

This persistent localized roaming behavior introduces distinct considerations regarding the long-term robustness of the whole-pond abundance extrapolation model. The mathematical validity of the global abundance inversion relies entirely on the structural stability of the horizontal and vertical distribution coefficients. Because these parameters quantify specific spatial utilization patterns, any disruption to behavioral uniformity directly threatens model accuracy. Recent advanced sonar monitoring frameworks highlight that fish vertical migration and spatial distribution profiles exhibit significant temporal shifts driven by routine feeding schedules and time-of-day dynamics [19,39].

Consequently, diurnal changes in ambient light, feeding schedules, and sudden stress responses induce rapid alterations in schooling density and vertical positioning, leading to temporal drift in the distribution coefficients. Under extreme operational scenarios or environmental fluctuations, reliance on static coefficient inputs increases the risk of systematic estimation bias. Furthermore, acoustic environments with high-density fish populations are inherently prone to severe target occlusion and motion blur, complicating automated regression and long-term tracking [40,41]. Maintaining model resilience over extended production cycles requires the progression from static parameterization to adaptive coefficient modeling informed by real-time environmental inputs.

A pertinent operational consideration in commercial aquaculture monitoring involves the validity of reference stocking baselines and spatial sampling protocols. In large-scale intensive aquaculture, lethal destructive harvesting following non-invasive acoustic trials is economically unviable and impairs fish welfare. By executing our acoustic surveys immediately after standardized nursery batch stocking, this study established an authentic baseline free from cumulative mortality, size grading, or split transfers. Furthermore, addressing spatial sampling geometry, our framework provides a practical resolution to the classic sampling dilemma between vertical beam spread and discrete depth scanning in shallow aquaculture facilities. Instead of attempting complete, non-overlapping acoustic insonification—which is technically unfeasible in shallow water columns given finite transducer beam angles—the decoupling of behavioral stratification profiling from volumetric density scaling ensures robust abundance inversion. This modular formulation demonstrates that standard narrow-aperture imaging sonars can achieve reliable whole-facility population quantification without requiring expensive multi-transducer arrays or complex tomographic inversions.

### 4.3. Limitations and Future Research

While rigorous, census-referenced quantitative validation in this study was primarily concentrated on juvenile largemouth bass in land-based recirculating aquaculture systems, the open-sea engineering demonstration was qualitatively extended to golden pompano in deep-sea net cages. Broader multi-species applicability across varying teleost morphologies and turbulent marine hydrodynamic regimes warrants continued empirical investigation. The foundational physical–statistical mechanics, specifically the Poisson occlusion correction governed by morphological eccentricity and the spatial extrapolation driven by horizontal and vertical coefficients, are mathematically species-agnostic. Consequently, extending this framework to alternative cultured species represents a process of populating a species-specific parameter library rather than a methodological redesign. Furthermore, this study deliberately focuses on numerical abundance estimation as the primary sensing objective without incorporating empirical length-to-weight conversions. Future research will focus on expanding this parameter database across diverse teleost morphologies, to directly convert size-resolved abundance into harvest biomass, and implementing multi-transducer arrays to dynamically update spatial coefficients under varying environmental conditions.

## 5. Conclusions

This study establishes an integrated acoustic monitoring framework combining YOLOv11 object detection, Poisson distribution statistical correction, and a spatial distribution coefficient expansion model to resolve target occlusion and limited detection range challenges in acoustic abundance estimation. Validated across controlled stocking abundance gradients from 20 to 100 individuals, the methodology achieves an 84.56% reduction in image noise and decreases visual detection errors by up to 75.83%. Commercial pond application demonstrates that global estimation errors are significantly reduced to a range between 1.38% and 16.43% (with an average error below 10%), representing up to a 96.44% reduction in error magnitude while simultaneously satisfying frame-level real-time processing requirements. By coupling deep-learning object detection with statistical spatial modeling, this work provides a non-invasive acoustic sensing methodology that effectively reduces occlusion-induced counting biases and sampling mismatches, providing reliable population estimates for high-density aquaculture management.

## Figures and Tables

**Table 1:** Technical Parameters of Imaging Sonar.

Parameter	750 kHz	1.2 MHz
Horizontal beam angle (°)	130	80
Vertical beam angle (°)	20	12
Maximum detection range (m)	120	40
Number of beams	512	512

**Table 2:** Evaluation of YOLOv11 detections and Poisson-based corrections under different stocking abundance gradients.

Stocking Density Gradient (ind.) ^1^	YOLOv11 Observed Mean (ind.)	YOLO Relative Error (%)	Cascaded Corrected Abundance (ind.)	Post-Correction Relative Error (%)	Error Reduction Efficiency (%)
20	7.78	61.10	16.50 ± 2.01	17.50	71.36
40	24.15	39.63	36.17 ± 1.12	9.58	75.83
60	36.48	39.20	51.83 ± 1.34	13.62	65.25
80	42.67	46.66	59.52 ± 1.27	25.60	45.14
100	45.69	54.31	62.78 ± 1.35	37.22	31.47

^1^ ind. stands for individuals. Raw YOLOv11 observed mean represents the average detection count within the 2D sonar slice. Cascaded corrected abundance represents the restored total enclosure population obtained by coupling micro-scale Poisson slice correction (Equation (13)) with effective vertical beam occupancy expansion $\eta_{v}$ (Equation (16), where $\eta_{v}$ ranges from 0.475 at low density to 0.765 at high density, reflecting behavioral vertical stratification). Relative error is evaluated against the true controlled stocking gradient. Values following ± indicate 95% confidence intervals derived from temporal frame variance and error propagation (Equation (14)).

**Table 3:** Validation results of the estimation framework in commercial-scale aquaculture ponds (Pond A and Pond B).

Pond	Trial	Valid Frames (N)	Per-Frame Raw Mean ${\bar{n}}_{yolo}$ (ind./frame)	Uncorrected Whole-Pond Baseline $N_{raw}$ (ind.)	YOLOv11 Relative Error (%)	Post-Correction Mean (ind.)	Post-Correction Relative Error (%)	Error Reduction Efficiency (%)
Pond A	1	402	13.39 ± 5.21	2813	53.12	5014	16.43	69.07
	2	420	15.93 ± 5.19	3347	44.22	5650	5.83	86.82
	3	418	14.44 ± 4.97	3034	49.43	5277	12.05	75.62
Pond B	1	312	18.94 ± 5.98	3980	38.77	6410	1.38	96.44
	2	371	17.08 ± 5.62	3588	44.80	5940	8.62	80.76
	3	415	15.40 ± 5.11	3235	50.23	5516	15.14	69.86

Note: The Uncorrected Baseline $N_{raw}$ represents the whole-pond population extrapolated directly from per-frame raw YOLOv11 detections ($n_{yolo}$) using the global extrapolation factor (≈210.14) without applying acoustic occlusion and layer-occupancy corrections. Post-correction abundance $N_{corr}$ is derived by cascading the in-slice Poisson model ($n_{slice}$), core vertical layer-occupancy recovery ($n_{local}=n_{slice}/\eta_{z}$, where $\eta_{z}\approx 0.569$), and the identical spatial extrapolation factor ($S_{extrap}$). This isolates the net recovery achieved by acoustic geometric calibration under consistent spatial scaling conditions.

**Table 4:** Acoustic monitoring parameters and estimated fish abundance across commercial offshore deep-sea net cages stocking golden pompano (*Trachinotus ovatus*).

Net Cage ID	Mean Fish Length (cm)	Mean Projected Area (cm^2^)	Mean Surface Coverage (%)	Sonar-Estimated Abundance (ind.)	Farm Stocking Baseline (ind.)
Cage 1	31.83±2.14	136.18±18.25	21.49	65,269	~60,000–70,000
Cage 2	34.62±2.30	158.70±20.12	24.74	61,536	~60,000–70,000
Cage 3	34.33±2.05	155.94±19.50	24.43	56,794	~60,000–70,000
Cage 4	35.54±2.68	169.83±22.40	32.20	98,220	~80,000–90,000

Note: Mean surface coverage denotes the average percentage of the 2D sonar sector area occupied by segmented acoustic fish echoes. Farm Stocking Baselines are commercial batch-stocking records provided by the mariculture facility management, representing macroscopic reference intervals that do not account for dynamic natural mortality or net-escape events during the open-sea grow-out phase.

## Data Availability

The data used to support the findings of this study are available from the corresponding author upon request.
